# Measuring cognitive insight in schizophrenia and bipolar disorder: a comparative study

**DOI:** 10.1186/1471-244X-7-71

**Published:** 2007-12-11

**Authors:** John A Engh, Svein Friis, Astrid B Birkenaes, Halldóra Jónsdóttir, Petter A Ringen, Torleif Ruud, Kjetil S Sundet, Stein Opjordsmoen, Ole A Andreassen

**Affiliations:** 1 Division of Psychiatry, Ulleval University Hospital, Oslo, Norway; 2 Institute of Psychiatry, University of Oslo, Norway; 3 Institute of Psychology, University of Oslo, Oslo, Norway; 4 Akershus University Hospital and University of Oslo, Oslo, Norway

## Abstract

**Background:**

Beck Cognitive Insight Scale (BCIS) has been designed for assessment of self-reflection on patients' anomalous experiences and interpretations of own beliefs. The scale has been developed and validated for patients with schizophrenia. We wanted to study the utility of the scale for patients with bipolar disorder. The relationship between the BCIS as a measure of cognitive insight and established methods for assessment of insight of illness was explored in both diagnostic groups.

**Methods:**

The BCIS self-report inventory was administered to patients with schizophrenia (n = 143), bipolar disorder (n = 92) and controls (n = 64). The 15 items of the inventory form two subscales, *self-reflectiveness *and *self-certainty*.

**Results:**

The internal consistency of the subscales was good for the patient groups and the controls. The mean subscale scores were not significantly different for the three groups. Four items in subscale self-reflectiveness referring to psychotic experiences gave, however, different results in the control subjects. Self-certainty and scores on insight item PANSS correlated significantly in the schizophrenia, but not in the bipolar group.

**Conclusion:**

BCIS with its two subscales seems applicable for patients with bipolar disorder as well as for patients with schizophrenia. The self-report inventory can also be applied to control subjects if the items referring to psychotic experiences are omitted. In schizophrenia high scores on self-certainty is possibly associated with poor insight of illness. For the bipolar group the subscales are largely independent of traditional insight measures.

## Background

Insight of illness is an important aspect in diagnostics and treatment of patients with severe mental disorders. Focus has primarily been on awareness of illness and treatment needs based on the widely used definition by Anthony David [1]. Aaron T. Beck labeled this *clinical insight *[2]. In addition to attenuated clinical insight, patients with psychosis often have reduced capacity to reflect rationally on their anomalous experiences and to recognize that their conclusions are incorrect [2]. Beck termed such insight *cognitive insight*. He summarized the relevant components of this concept as "impairment of objectivity about the cognitive distortions, loss of ability to put these into perspective, resistance to corrective information from others and overconfidence in conclusions [2]."

To measure cognitive insight, he developed the Beck Cognitive Insight Scale (BCIS), a 15 item self-report instrument with two subscales, self-reflectiveness and self-certainty (Beck 2004). Acceptable levels of internal consistency (alpha self-reflectiveness 0.68, alpha self-certainty 0.60) were found for a mixed sample of patients with schizophrenia, schizoaffective disorder and major depression [2]. For middle-aged and older patients with schizophrenia and schizoaffective disorder, acceptable internal consistency was later confirmed for self-reflectiveness (alpha 0.70), but not for self-certainty (alpha 0.50) [3].

The introduction of the BCIS has provided an opportunity to explore cognitive insight among patients with schizophrenia. Beck and colleagues recruited 150 inpatients, 75 diagnosed with schizophrenia and schizoaffective disorder and 75 with major depressive disorder. Twenty-one per cent in the latter group had psychotic depression. The authors [2] showed that the subscale self-certainty differentiated between major depressive patients with and without psychosis. However, the BCIS has not yet been validated for patients with bipolar I and bipolar II disorder, in whom lack of insight may also be a major clinical problem. We have earlier demonstrated that a self-report questionnaire assessing insight of illness with good psychometric properties for patients with schizophrenia may not necessarily function as well for patients with bipolar disorders [4]. It is therefore important to study the psychometric qualities of the BCIS also for this patient group. Self-reflectiveness and self-certainty comprise items that seem to be rather general in content. Possibly, the two subscales cover a spectrum from normality to pathology. Warman *et al.*[5] have recently published an article which points out that the factor loadings and internal consistencies of the BCIS were similar for healthy controls and the two groups of inpatients in Beck's original paper. We intended to replicate the investigation of the subscales' utility for normal controls.

The aims of the present study were: 1) to examine the subscale scores, internal consistency and intercorrelation of the BCIS' subscales for the schizophrenia group, the bipolar group and for normal controls, 2) to explore the relationship between the BCIS and affective symptom scores, and established scales for assessment of insight for the two diagnostic groups, and 3) to compare mean scores for patients and controls.

## Methods

The subjects participated in a large ongoing study on schizophrenia and bipolar disorders (TOP Study, Thematic Organized Psychoses Research) and were recruited from out-patient and in-patient psychiatric units at four University Hospitals in Oslo, Norway, from March, 2005 through July, 2007. The health care system is catchment area based and the patients are referred from primary care. The patients were invited to participate in the study by the clinician responsible for their treatment.

All participants gave written informed consent, and the study was approved by the Regional Committee for Medical Research Ethics and the Norwegian Data Inspectorate.

### Participants

The inclusion criteria were as follows: age 18 to 65 years, able to understand and speak a Scandinavian language, meeting the DSM-IV criteria for schizophrenia, schizoaffective, schizophreniform or bipolar disorder, no history of severe head trauma, IQ score of above 70 and willing and able to give informed consent. A total of 235 patients met the criteria. The schizophrenia group consisted of 143 patients with the DSM-IV diagnoses schizophrenia (n = 107), schizophreniform disorder (n = 10), and schizoaffective disorder (n = 26). Ninety-two patients were included in the bipolar group, diagnosed with either bipolar I disorder (n = 45), bipolar II disorder (n = 43) or bipolar NOS (n = 4). Sixty-four healthy subjects took part in the control group. Twenty-seven of these individuals were randomly selected from statistical records from the same catchment area as the patients. The remaining 37 persons were a mixed group of volunteering professionals. Detailed analyses of the subscale scores and intra class correlation showed similar results in the two control samples, and hence we refer only to one control group in this article.

Diagnosis was established using the Structured Clinical Interview for DSM-IV-TR-axis I disorders (SCID-I) [6]. All interviewers finished a training course in SCID assessment based on the training program at UCLA [7] and participated in diagnostic evaluation meetings on regular basis led by a clinically experienced professor in psychiatry (S.O.). Mean overall kappa for SCID diagnoses assessed by the UCLA was 0.77 (95 % CI: 0.60–0.94).

### Measures

#### General assessments

History of mental illness, present symptoms, life style, and pharmacological treatment were obtained from interview with the patient, with additional information collected from treatment records and clinical staff. Severity of symptoms was assessed by the Positive and Negative Syndrome Scale PANSS (PANSS) [8], the Young Mania Rating Scale-Clinician rated (YMRS-C) [9] and the Inventory of Depressive Symptoms-Clinician rated (IDS-C) [10]. The assessment of depression was conducted partly with the Inventory of Depressive Symptoms-Clinician rated (IDS-C), partly with CDSS (Calgary Depression Scale), but only the former scores are utilized in the study. Hence, there is a discrepancy between the number of patients with IDS scores and the number of patients with other symptom scores (PANSS, YMRS). Psychosocial functioning was measured by the Global Assessment of Functioning Scale (GAF) [11,12], and the scores were split into scales of symptoms (GAF-S) and function (GAF-F) to improve psychometric properties. The inter-rater reliability of the investigators was good for the GAF with an intra class correlation, ICC 1.1, of 0.86 [13].

#### Assessment of cognitive insight and insight of illness

**Figure 1:**
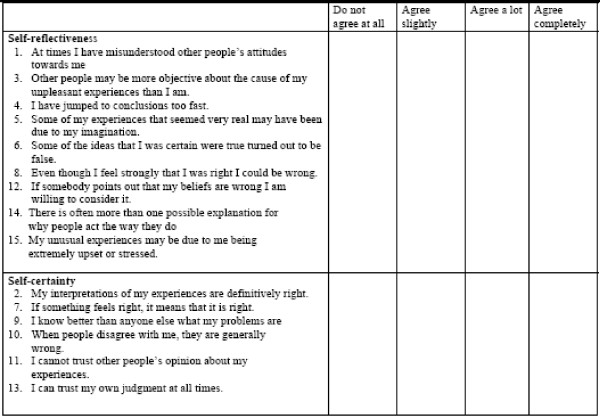
BCIS subscales.

The BCIS is a self-report inventory consisting of 15 statements rated on a 4-point Likert scale (0 = do not agree at all to 3 = agree completely). Based on factor analyses Beck and coworkers divided the 15 items into 2 subscales (Figure 1). The first component consisted of 9 items measuring objectivity, reflectiveness and openness to feedback and given the label *self-reflectiveness*. Under the umbrella of decision-making and resistance to feedback, 6 items were united in a second component of the scale, labeled *self-certainty*. High scores on the subscale self-reflectiveness and low scores on subscale self-certainty is considered as normal. With approval from the authors the inventory was translated from English into Norwegian and the procedure was reversed under blinded conditions back into English. The questionnaire was administered without a time limit.

Insight of illness was assessed for both diagnostic groups by the PANSS insight item (G12) [14]. A score of three or higher was defined as poor insight of illness.

### Patient characteristics

Table 1 gives an overview of demographic and clinical characteristics for the two patient groups. The proportion of men in the schizophrenia group was significantly larger than in the control group. Subjects in the schizophrenia group were significantly younger than the bipolar patients. Additionally, the subjects in the schizophrenia group had significantly higher scores on symptom measures (PANSS delusions, PANSS hallucinations, PANSS pos., PANSS neg., PANSS total) and also larger variability than in the bipolar group. Sixty-four of the patients in the schizophrenia sample (44.8 per cent) obtained scores above cut off (4 or higher) on items PANSS delusions and/or PANSS hallucinations, where as only 4 patients in the bipolar sample (4.3 per cent) attained scores above cut off. Similarly GAF-S and GAF-F were significantly lower in the schizophrenia group than in the bipolar group, with mean scores in the schizophrenia group reflecting a symptom level bordering psychosis. The variance for both GAF-measures was lower in the schizophrenia group. The mean score on G12 was significantly higher in the schizophrenia group than in the bipolar group, pointing toward poorer insight of illness in the former group. Fifty per cent of the patients in the schizophrenia group and 14 per cent of the bipolar patients obtained G12 scores reflecting poor insight of illness.

**Table 1:** Demographic and clinical variables

	**Schizophrenia (1) n = 143**	**Bipolar disorder (2) n = 92**	**Controls (3) n = 64**	**1 vs. 2**	**1 vs. 3**	**2 vs. 3**
**Male**	83 (58.0)	40 (43.5)	25 (39.1)	n.s	<0.05	n.s.
**Age**	32.5 (9.5)	37.2 (11.4)	33.7 (11.7)	<0.05	n.s.	n.s.
**GAF-S**	42.8 (11.4)	61.2 (12.1)		<0.001		
**GAF-F**	43.8 (10.9)	61.6 (14.3)		<0.001		
**PANSS delusions**	3.0 (1.7)	1.4 (0.9)		<0.001		
**PANSS hallucinations**	2.6 (1.7)	1.2 (0.8)		<0.001		
**PANSS pos**	14.7 (5.9)	9.1 (2.9)		<0.001		
**PANSS neg**	14.4 (5.5)	10.1 (3.7)		<0.001		
**PANSS total**	58.3 (16.6)	43.6 (10.9)		<0.001		
**IDS-C total**	17.0 (12.5)	15.8 (12.6)		n.s.		
**PANSS insight**	2.6 (1.5)	1.5 (1.0)		<0.001		

Male, per cent in parenthesis; for other variables, standard deviation in parenthesis; GAF-S, Global Assessment of Functioning – Symptom scale; Global Assessment of Functioning – Function scale; PANSS, Positive and Negative Syndrome Scale, delusions item, hallucinations item, positive symptoms subscale, negative symptoms subscale, total score; IDS-C, Inventory of Depressive Symptoms Scale-Clinician and PANSS insight item.

### Statistics

For the statistical analysis we used SPSS version 13. Student-t-test was utilized for examining statistical significance between the two patient groups on clinical variables. ANOVA and post-hoc tests were used when comparing all three groups on age and gender distribution, in addition to comparison of BCIS scores.

Few patients had missing data on the BCIS. The missing items were scattered on the various items in both subscales. On the average there were 1.5 total missing registrations per item for each of the patient groups. For the controls there was a clear difference between the subscales, as 14.3 % had omitted self-reflectiveness items, but only 3.1 % had left out self-certainty items. For PANSS total and YMRS total score there were 2 and 1 missing registrations respectively in the schizophrenia sample and 1 and 4 in the bipolar sample.

The internal consistency for the two subscales was calculated as Cronbach's alpha for the two diagnostic groups and for the controls. We followed the recommendations by Nunally that a satisfactory alpha equals or is higher than 0.70 [14].

The average correlation between each item and the remaining items within the subscale (corrected item total correlation, CITC) was also calculated. Furthermore, we computed the subscale intercorrelation for the different diagnostic groups. Finally, self-reflectiveness and self-certainty of the BCIS were correlated with scores on the G12 using the Pearson correlation test.

## Results

### Psychometric properties and scale scores of the BCIS

As shown in Table 2 alpha for self-reflectiveness was the same for all groups. Alpha for self-certainty, displayed in Table 3, was somewhat lower, but equally consistent across groups.

**Table 2:** Internal consistency

	**Schizophrenia **n = 143		**Bipolar disorder **n = 92		**Controls **n = 64	
	Cronbach's alpha	CITC	Cronbach's alpha	CITC	Cronbach's alpha	CITC

**Self-reflectiveness**	0.72	0.39	0.73	0.40	0.73	0.41
**Self-certainty**	0.63	0.36	0.61	0.35	0.63	0.38

CITC, Corrected Item Total Correlation

**Table 3:** Subscale scores

	**Schizophrenia (1)**			**Bipolar disorder (2)**			**Controls (3)**			**1 vs. 2**	**1 vs. 3**	**2 vs. 3**
	N	Mean	S.D.	N	Mean	S.D.	N	Mean	S.D.	*p*	*p*	*p*

**Self-reflectiveness**	143	14.5	4.8	92	14.7	4.7	64	13.4	4.3	n.s.	n.s.	n.s.
**Self-certainty**	143	8.2	3.4	92	7.4	3.0	64	8.0	2.7	n.s.	n.s.	n.s.

S.D., standard deviation.

We found no significant difference in mean scores for self-reflectiveness or self-certainty in any of the groups. Additionally, both subscales showed similar variance in all groups.

### BCIS subscales divided according to content

The similar subscale scores attained in all groups was unexpected. An obvious question would be if the statements in the various items were understood differently by the groups. The high percentage of self-reflectiveness items omitted by the controls indicated that the subscale contained items which were conceived differently among these subjects than among the patients. We found that item 3, 5, 6 and 15 were left out to a large degree and that these items consisted of statements which could be interpreted as referring to psychotic experiences and, therefore difficult to answer by the control subjects. Self-reflectiveness was split into *component I*, consisting of these pathological experiences, and *component II *comprising the remaining items. As shown in Table 4 the mean score for component I was significantly lower for the controls than for both the schizophrenia and the bipolar group. The correlations between the mean scores of the two components were 0.55, 0.40 and 0.52 for the schizophrenia group, the bipolar group and controls respectively, indicating moderate to strong relationships.

**Table 4:** Self-reflectiveness divided in two components.

	**Schizophrenia (1)**			**Bipolar disorder (2)**			**Controls (3)**			**1 vs 2**	**1 vs 3**	**2 vs 3**
	**N**	**Mean pr item**	**SD**	**N**	**Mean pr item**	**SD**	**N**	**Mean pr item**	**SD**	***p ***	***p***	***p***

**Component I**	143	1.34	0.72	92	1.19	0.72	64	0.86	0.64	n.s.	<0.001	<0.05
**Component II**	143	1.83	0.52	92	1.99	0.55	64	1.98	0.48	n.s.	n.s.	n.s.

Component I, items referring to psychotic experiences; Component II, remaining items.

### Relationship between scores on BCIS and psychopathology

There was no significant association between self-reflectiveness and YMRS total score or between self-reflectiveness and IDS total score in schizophrenia. For Self-certainty, however, we found a significant relationship to YMRS total score in this group. We did not find a significant association between any of the BCIS subscales and these measures of affective symptoms in the bipolar group. The relationship between the BCIS subscales and PANSS positive in the bipolar group was also nonsignificant.

Sixteen of the subjects diagnosed with bipolar disorder reported one or more previous psychotic episodes. These subjects attained scores on component I and II which were similar to the scores in the group of remaining subjects in the bipolar sample.

We wanted to investigate if there was a difference in self-reflectiveness and self-certainty scores between inpatients and outpatients. In a subsample of 78 schizophrenia patients no difference was found on self-reflectiveness or self-certainty scores. There were very few hospitalized bipolar patients and hence the comparison was unfeasible.

### Comparing BCIS and measures of insight

As displayed in Table 5 and 6 the correlation between self-reflectiveness and self-certainty was low for the schizophrenia group and significant for the bipolar group.

**Table 5:** Correlation coefficients between scores of cognitive insight and insight of illness for schizophrenia.

	**Self-reflectiveness**	**Self-certainty**
**Self-certainty**	-0.13	
**PANSS insight**	-0.21*	0.38**

*p < 0.05; ** p < 0.01.

**Table 6:** Correlation coefficients between scores of cognitive insight and insight of illness for bipolar disorder.

	**Self-reflectiveness**	**Self-certainty**
**Self-certainty**	-0.21*	
**PANSS insight**	-0.03	0.15

*p < 0.05

We calculated the correlation between self-reflectiveness, self-certainty and G12 score and found a highly significant positive correlation between self-certainty and G12 for schizophrenia.

## Discussion

The main finding was that the psychometric properties of both subscales of the BCIS were acceptable for the schizophrenia and for the bipolar group. The scores of the controls, however, can not be compared to patient scores without excluding items referring to psychotic experiences. Furthermore, the two subscales self-reflectiveness and self-certainty showed low or moderate correlation for the three groups, indicating that they represent two different dimensions of cognitive insight.

Internal consistency for each subscale of the BCIS was consistent across groups, somewhat higher for self-reflectiveness than for self-certainty. Previous findings for the schizophrenia group [2,3] were thereby replicated. These findings are also in line with the results by Warman *et al.*[5] which point out that the factor loadings and internal consistencies of the BCIS were similar for healthy controls and the two groups of inpatients in Beck's original paper. To our knowledge the psychometric properties of the BCIS have not previously been published for a bipolar sample.

The psychometric properties of the BCIS were similar for normal controls to what we found in schizophrenia and bipolar disorder. However, the self-reflectiveness scores in the two patient groups were actually higher than in the control group, although not statistically significant.Similar findings concerning scores on subscale self-reflectiveness was found in a recent study by Warman *et al.*[5] comparing scores of undergraduate students with patients diagnosed with schizophrenia or schizoaffective disorder. In this study the controls scored non-significantly lower on self-reflectiveness and significantly lower on self-certainty than subjects in the schizophrenia group. Similarly, in a study by Eric Granholm (personal communication) middle aged and older controls obtained self-reflectiveness and self-certainty scores that were lower than the patient scores in the Beck study.

We wanted to investigate if different scoring profiles were present in the three groups. Comparing scores across these groups on component I which includes "unusual experiences" disclosed a significantly lower score for controls than both patient groups. This discrepancy indicates that the control subjects interpret the statements of these particular items rather differently from the patients, which is understandable due to lack of psychotic experiences in this group. Control subjects and patients seem to have a different reference point for 4 out of the 9 items in self-reflectiveness. This implies that comparison of scores between control subjects and patients should be carried out only for component II. On the other hand, the two components within self-reflectiveness were fairly strongly correlated for all groups, and consequently, there was no clear indication that they constitute two separate dimensions. Provided that the scores of the controls are not compared with patients whose interpretation of items involving "unusual experiences" are likely to be different, all items in subscale self-reflectiveness might be applicable for controls for means of investigating relations between cognitive insight and other domains such as psychopathology.

We also investigated the scores on each of these components in our bipolar sample and found that the patients with and without previous psychotic episodes did not come out differently. This could be considered as additional support to the applicability of the scale in bipolar samples.

Self-reflectiveness and self-certainty were weakly or moderately intercorrelated in the groups suggesting that they represent different dimensions. Self-certainty showed a highly significant positive correlation with G12 scores in the schizophrenia group, indicating that mental inflexibility is possibly associated with poor clinical insight of illness. For self-reflectiveness a significant negative correlation was found for this group, suggesting that the capacity to reflect on anomalous experiences is linked to insight of illness. Further exploration of self-certainty could expand our knowledge of cognitive processes involved in insight of illness in general, and in particular comprehending patients' lack of insight related to treatment need. The relationship between poor mental flexibility and both symptoms and cognition are not known, and investigating these associations could represent important topics for future studies.

## Conclusion

The Beck Cognitive Insight Scale (BCIS) with its two subscales self-reflectiveness and self-certainty seems applicable for patients with schizophrenia and bipolar disorders. The BCIS can also be applied to control subjects, but in the case of comparison between controls and other clinical samples the four items referring to psychotic experiences should not be used. Our findings indicate that self-reflectiveness and self-certainty represent independent domains. In schizophrenia high self-certainty and low self-reflectiveness seem to be associated with poor insight of illness.

## Competing interests

The author(s) declare that they have no competing interests.

## Authors' contributions

JAE, HJ, ABB and PAR recruited patients to the study and carried out the clinical testing. SF, SO, KSS, TR and OAA participated in the design and coordination of the study. Statistical analyses were conducted by JAE, SF and OAA. All authors participated in the writing and all authors read and approved the final manuscript.

## Pre-publication history

The pre-publication history for this paper can be accessed here:


